# Supplementary material for: The associations between resilience, self-care, and burnout among medical students
Source: PLoS One. 2024 Sep 19;19(9):e0309994. doi: 10.1371/journal.pone.0309994 (PMC11412677; doi:10.1371/journal.pone.0309994)
Supplement: S1 File — (PDF) [file pone.0309994.s001.pdf]

The study fundamental data

1	4	2	4	2	4	2	2	3	2	3	1	3	2	4	4	4	5	4	3
	5	5	4	3	4	4	3	4	2	1	3	1	2	3	5	4	4	4	4
	4	4	4	4	5	4	4	4	3	5	4	5	3	2	2	4	1	4	1
	1	3	3	4	2	1	1												
2	5	1	5	2	5	2	1	3	1	5	1	1	2	5	5	5	5	5	4
	5	5	4	5	4	2	1	3	2	1	2	1	1	4	2	3	3	4	5
	5	5	5	5	3	3	2	5	4	5	5	5	3	3	3	5	5	2	2
	1	3	1	5	1	1	1												
3	1	5	1	5	1	5	5	5	3	3	2	1	1	5	3	4	3	2	2
	3	3	3	2	2	2	2	2	1	1	2	1	1	2	2	2	2	2	3
	3	3	3	3	3	3	1	3	3	2	1	3	1	1	3	1	2	1	2
	1	2	1	3	1	1	1												
4	5	1	4	3	4	2	5	5	5	3	3	3	1	5	3	4	4	3	2
	4	5	4	4	4	3	2	2	1	3	2	3	3	1	1	1	1	3	4
	5	5	5	3	2	2	3	3	2	2	4	4	2	2	4	3	3	3	2
	1	2	1	2	2	1	1												
5	5	2	4	2	2	2	2	3	3	4	2	2	2	4	3	4	4	4	3
	4	4	4	4	3	2	2	4	3	2	3	1	2	4	4	4	4	4	5
	5	4	4	3	3	3	3	3	3	4	4	4	3	2	2	4	1	3	1
	1	2	9	4	1	1	1												
6	4	4	3	3	2	2	2	4	3	2	3	4	3	2	4	3	2	3	3
	1	3	3	4	3	4	4	3	4	1	4	1	4	4	4	4	4	4	4

	4<br>1	5<br>3	3<br>9	2<br>5	3<br>1	4<br>1	2<br>3	4	3	4	3	4	1	3	1	2	1	3	2
7	5<br>4<br>4<br>1	2<br>4<br>4<br>1	2<br>4<br>4<br>9	2<br>4<br>4<br>4	4<br>4<br>3<br>2	3<br>4<br>4<br>1	3<br>2<br>3<br>1	4<br>3<br>4	3<br>4<br>3	3<br>2<br>4	3<br>2<br>4	4<br>1<br>4	2<br>1<br>2	4<br>1<br>2	4<br>1<br>3	4<br>1<br>4	4<br>1<br>1	4<br>4<br>3	4<br>4<br>2
8	4<br>5<br>4<br>1	3<br>5<br>3<br>1	4<br>4<br>3<br>9	3<br>5<br>3<br>4	4<br>5<br>3<br>1	2<br>4<br>3<br>3	1<br>2<br>2<br>1	1<br>2<br>4	1<br>1<br>2	4<br>2<br>3	1<br>1<br>3	1<br>1<br>5	1<br>1<br>3	4<br>4<br>5	4<br>4<br>5	5<br>5<br>5	5<br>5<br>5	3<br>5<br>2	5<br>3<br>2
9	4<br>5<br>4<br>1	3<br>4<br>4<br>3	4<br>4<br>4<br>9	2<br>4<br>5<br>4	4<br>4<br>4<br>2	2<br>2<br>4<br>1	3<br>2<br>4<br>1	2<br>3<br>4	2<br>2<br>5	4<br>2<br>5	1<br>4<br>5	2<br>3<br>5	2<br>2<br>3	5<br>2<br>2	4<br>3<br>5	4<br>3<br>4	4<br>3<br>3	4<br>4<br>4	4<br>5<br>2
10	4<br>5<br>5<br>1	2<br>5<br>5<br>3	4<br>5<br>5<br>9	2<br>5<br>5<br>5	4<br>5<br>3<br>2	2<br>1<br>4<br>1	1<br>3<br>2<br>1	3<br>5<br>5	1<br>2<br>5	5<br>1<br>5	1<br>3<br>5	3<br>1<br>5	1<br>1<br>3	5<br>1<br>2	5<br>2<br>2	5<br>2<br>3	5<br>2<br>1	5<br>5<br>4	5<br>5<br>2
11	2<br>4<br>5<br>1	4<br>4<br>4<br>3	2<br>4<br>4<br>9	5<br>3<br>2<br>3	2<br>3<br>3<br>2	4<br>3<br>3<br>1	4<br>3<br>2<br>3	3<br>2<br>5	3<br>3<br>1	5<br>2<br>4	1<br>1<br>4	1<br>1<br>4	1<br>2<br>4	5<br>1<br>3	4<br>1<br>3	5<br>1<br>2	4<br>1<br>1	3<br>4<br>2	2<br>5<br>2
12	5<br>4	2<br>5	4<br>4	2<br>5	4<br>4	2<br>4	3<br>1	4<br>2	4<br>2	2<br>2	2<br>2	2<br>1	2<br>3	4<br>4	2<br>4	5<br>3	4<br>4	5<br>4	3<br>5

	4	3	4	4	4	3	2	4	2	4	5	3	1	2	1	4	5	4	2
	1	1	9	5	2	1	1												
13	5	2	4	2	4	4	2	4	1	5	1	3	1	5	5	4	3	4	4
	3	4	5	5	4	3	2	2	3	2	2	1	1	4	3	4	3	4	5
	5	5	5	5	2	3	3	5	4	4	5	5	4	2	3	4	1	3	2
	2	2	9	3	2	1	1												
14	5	1	4	1	4	3	2	3	2	4	3	3	1	5	4	3	4	4	4
	5	4	4	4	4	3	2	3	4	1	4	1	1	1	4	3	3	4	5
	5	5	4	5	3	4	4	3	3	5	3	4	1	1	3	4	1	3	1
	1	2	9	3	2	1	1												
15	4	2	2	3	4	3	3	4	3	3	3	2	2	2	4	4	4	4	4
	4	4	4	4	3	2	2	4	3	2	4	1	1	3	5	5	5	4	5
	5	4	5	5	4	4	5	5	5	4	5	4	2	1	4	3	1	4	2
	1	2	9	3	2	1	1												
16	5	1	5	1	1	2	1	2	1	4	2	2	1	3	3	3	5	5	3
	3	5	5	5	5	3	2	4	3	1	3	1	1	2	1	2	2	5	5
	5	5	5	4	2	4	3	5	4	5	4	5	2	1	1	2	1	4	1
	1	2	9	3	3	1	1												
17	4	3	4	3	2	4	2	3	3	4	3	2	3	4	3	4	4	3	2
	3	4	3	3	4	2	2	2	2	3	3	2	3	3	2	3	3	3	3
	5	4	4	3	4	3	2	4	3	3	3	4	2	4	1	4	1	3	1
	1	3	2	3	2	1	1												
18	4	2	3	2	4	2	4	4	2	4	1	2	2	4	3	4	4	3	3
	4	4	4	4	4	2	3	3	3	3	3	1	2	3	4	3	3	3	4

	3	4	3	3	3	4	3	4	3	3	4	3	3	2	3	4	1	3	1
	1	1	9	4	2	1	1												
19	5	2	4	2	4	3	1	4	3	1	3	2	3	3	3	4	4	4	3
	3	3	3	5	4	4	4	2	2	1	4	1	3	2	2	2	3	3	3
	4	4	3	3	3	4	5	4	3	5	4	4	2	2	2	3	1	4	2
	1	2	9	4	2	1	1												
20	5	2	4	2	4	2	2	3	2	2	2	2	3	3	3	4	4	4	4
	4	4	4	4	4	3	3	4	3	3	4	3	2	3	3	3	3	4	2
	4	4	4	4	4	5	5	5	3	4	3	3	2	2	2	2	1	4	1
	1	3	1	3	3	1	1												
21	4	5	1	5	3	4	2	3	1	2	1	2	2	5	3	5	4	5	4
	4	5	5	5	4	4	4	2	2	1	4	1	1	1	3	4	4	3	4
	4	4	5	3	4	3	2	5	3	3	5	4	4	4	3	5	1	3	2
	2	3	9	4	1	1	2												
22	4	4	3	2	4	2	3	4	2	3	2	3	2	5	3	4	4	3	3
	3	4	4	4	3	3	4	4	4	3	4	4	2	3	4	4	3	3	3
	4	5	4	3	4	4	5	4	3	3	4	4	4	3	2	3	4	4	2
	1	3	9	4	1	1	1												
23	5	2	4	3	4	5	1	2	1	4	2	1	1	5	4	5	4	3	3
	5	4	3	3	3	3	1	2	1	2	2	1	1	1	1	1	1	4	4
	4	4	4	3	2	3	5	4	1	3	5	4	3	1	1	4	1	5	2
	1	2	9	4	2	1	1												
24	4	2	3	2	2	2	2	2	2	3	1	2	2	2	3	5	4	5	5
	5	5	4	5	5	3	3	3	4	3	3	1	2	4	3	4	3	4	5

	5	5	5	5	5	3	4	4	3	4	4	4	4	4	2	4	1	4	2
	1	3	9	4	2	1	3												
25	4	2	4	2	4	4	3	4	3	4	2	2	2	5	3	4	3	4	3
	4	4	4	4	4	2	2	3	2	2	4	2	2	2	2	2	2	4	4
	4	4	4	4	3	3	3	4	3	4	4	4	2	2	1	4	1	4	2
	1	3	2	4	2	1	1												
26	5	3	4	3	4	4	4	4	4	2	5	5	4	2	3	3	4	3	3
	3	3	4	4	3	4	4	3	3	2	3	1	2	1	5	2	1	3	3
	4	5	5	5	4	4	5	5	5	4	4	3	2	1	3	3	1	5	2
	1	3	9	4	2	1	2												
27	4	1	5	3	4	3	2	4	2	4	4	1	1	5	3	2	4	4	4
	4	4	3	3	2	1	2	2	2	1	2	1	1	1	4	3	2	4	3
	3	4	4	5	3	4	4	4	3	3	3	4	4	2	3	2	3	4	2
	1	1	9	5	2	1	1												
28	5	2	4	1	4	1	2	3	3	2	3	2	3	2	3	3	3	4	3
	4	3	3	3	2	2	3	3	1	1	2	1	3	1	4	1	1	4	4
	4	4	4	4	2	2	3	2	2	4	3	4	1	1	1	2	1	3	1
	1	1	9	2	3	1	1												
29	5	1	5	1	4	3	2	2	2	4	2	2	1	5	3	4	5	5	5
	5	5	3	5	3	3	3	3	3	3	3	1	3	2	4	2	3	4	4
	5	5	4	5	3	3	4	5	2	4	5	4	4	1	1	4	5	4	2
	1	1	9	2	2	1	1												
30	4	2	4	2	4	2	1	3	1	2	1	1	1	4	3	4	4	4	4
	3	4	4	2	3	3	3	3	2	2	4	2	3	3	2	3	3	3	3

	4<br>1	4<br>1	4<br>9	4<br>3	4<br>2	4<br>1	3<br>3	4	2	3	4	4	4	5	2	3	1	3	1
31	3<br>2<br>3<br>1	3<br>2<br>3<br>1	3<br>3<br>3<br>9	3<br>3<br>3<br>4	3<br>3<br>3<br>2	3<br>2<br>3<br>1	2<br>4<br>2<br>1	2<br>4<br>4	2<br>3<br>4	3<br>3<br>3	2<br>4<br>4	2<br>1<br>4	1<br>3<br>3	3<br>3<br>1	3<br>3<br>1	3<br>4<br>3	4<br>4<br>1	4<br>3<br>3	3<br>3<br>1
32	4<br>4<br>4<br>1	3<br>4<br>4<br>2	3<br>4<br>4<br>9	2<br>3<br>3<br>3	4<br>4<br>2<br>2	2<br>1<br>2<br>1	5<br>1<br>2<br>1	5<br>2<br>2	5<br>1<br>2	3<br>1<br>3	4<br>1<br>2	3<br>1<br>3	1<br>2<br>2	3<br>1<br>2	2<br>3<br>1	3<br>1<br>2	4<br>1<br>1	4<br>3<br>2	2<br>4<br>1
33	2<br>3<br>4<br>1	4<br>3<br>4<br>3	2<br>4<br>4<br>3	4<br>3<br>4<br>3	2<br>4<br>4<br>1	5<br>2<br>3<br>1	1<br>4<br>1<br>3	3<br>3<br>5	2<br>4<br>1	2<br>2<br>3	2<br>4<br>4	3<br>1<br>3	2<br>2<br>3	5<br>1<br>5	2<br>1<br>1	4<br>1<br>3	4<br>1<br>1	3<br>3<br>2	2<br>5<br>2
34	4<br>3<br>4<br>1	2<br>3<br>5<br>3	4<br>4<br>3<br>9	2<br>3<br>3<br>5	3<br>4<br>3<br>2	3<br>4<br>4<br>1	2<br>4<br>3<br>1	1<br>2<br>4	1<br>1<br>3	3<br>1<br>4	4<br>3<br>5	2<br>1<br>4	4<br>2<br>2	3<br>3<br>2	2<br>2<br>4	5<br>2<br>4	4<br>3<br>1	4<br>3<br>4	4<br>5<br>1
35	4<br>5<br>3<br>1	2<br>4<br>5<br>3	4<br>3<br>4<br>1	2<br>4<br>3<br>5	4<br>4<br>4<br>1	2<br>2<br>2<br>1	2<br>1<br>3<br>1	5<br>3<br>3	3<br>1<br>2	3<br>1<br>4	2<br>2<br>5	2<br>1<br>4	3<br>1<br>2	3<br>3<br>1	3<br>5<br>1	5<br>3<br>4	4<br>1<br>2	4<br>3<br>2	2<br>5<br>2
36	4<br>4	2<br>4	4<br>4	2<br>4	3<br>3	2<br>5	2<br>3	3<br>3	1<br>3	3<br>1	4<br>2	4<br>1	2<br>3	5<br>2	3<br>3	4<br>1	4<br>1	4<br>3	3<br>4

	4	4	5	3	1	2	3	4	2	4	4	4	1	1	2	2	1	4	1
	1	3	9	3	2	1	1												
37	5	2	5	2	4	2	2	3	1	4	2	2	1	4	4	3	4	4	5
	5	5	4	4	5	3	3	2	3	2	4	1	2	2	3	2	2	4	4
	4	5	4	3	3	5	3	5	5	3	4	5	3	5	3	5	1	3	1
	1	1	9	3	2	3	3												
38	5	2	5	2	4	2	2	3	2	3	1	2	2	4	3	4	4	5	4
	3	5	3	5	4	1	2	4	2	2	3	1	2	2	1	2	2	4	4
	4	4	3	4	2	4	5	4	3	4	5	4	3	1	5	2	4	2	2
	1	1	9	4	1	1	1												
39	5	2	5	2	4	2	2	5	3	3	1	1	2	2	3	3	5	2	2
	2	4	4	3	2	2	3	4	4	3	2	1	1	2	1	1	1	3	4
	5	4	4	2	2	2	2	3	2	4	3	5	5	3	1	3	1	4	1
	1	3	9	3	2	1	1	28											
40	5	2	5	2	4	2	2	3	1	5	2	1	1	5	5	4	4	5	3
	4	5	5	5	4	3	2	5	5	2	3	1	3	2	3	3	2	5	5
	5	5	5	5	4	4	3	5	5	5	5	5	3	1	4	3	1	5	1
	1	2	9	5	2	1	1	27											
41	5	2	4	2	4	2	1	4	2	4	2	2	2	3	3	4	4	4	4
	4	4	4	4	4	3	2	4	3	1	4	1	1	1	1	1	1	4	4
	4	4	4	4	1	4	3	4	3	4	4	4	1	1	3	4	1	3	1
	1	1	9	4	2	1	1	28											
42	5	1	5	1	5	1	1	2	1	3	1	2	1	5	3	4	5	5	4
	5	5	5	3	5	4	3	4	5	2	4	2	2	4	5	5	5	4	3

	4	4	4	5	2	5	5	5	5	5	5	5	2	2	3	4	1	2	2
	1	2	9	3	2	1	1												
43	5	2	4	2	3	2	3	3	4	2	4	3	2	3	2	4	4	4	4
	4	4	3	5	4	4	4	2	2	3	3	1	2	3	2	4	2	3	5
	3	4	4	3	3	4	3	4	3	3	4	5	1	1	1	4	1	4	2
	1	1	9	4	2	1	1	26											
44	5	2	4	2	4	2	3	3	2	4	2	2	2	3	4	3	4	4	4
	3	4	4	4	3	3	3	3	3	3	3	2	3	2	3	2	2	4	4
	4	4	4	3	3	4	3	4	3	3	3	4	3	2	2	3	2	3	2
	1	2	9	3	2	1	1	27											
45	3	2	4	3	2	3	3	2	2	3	2	3	2	4	3	4	4	4	4
	4	4	4	4	4	2	2	2	2	2	4	1	2	2	4	2	2	4	4
	4	3	3	4	4	4	4	4	3	4	4	4	3	3	2	4	2	3	2
	1	2	9	4	2	1	1	26											
46	5	2	5	1	5	2	2	4	4	5	3	2	2	4	3	4	5	4	3
	5	5	4	4	4	2	2	3	4	2	3	1	1	3	4	5	3	5	5
	5	5	5	4	2	3	3	3	3	5	5	5	4	2	1	3	1	5	2
	1	1	9	4	1	2	1	24											
47	4	4	2	4	1	3	2	2	2	3	3	2	2	4	2	5	4	5	5
	5	4	4	5	3	3	3	2	1	1	4	1	1	4	3	4	3	4	3
	3	4	4	4	4	5	2	5	4	3	4	3	1	1	4	4	1	3	2
	4	1	9	3	2	1	1	28											
48	5	2	4	3	3	3	2	3	2	4	3	4	1	4	2	5	5	4	4
	4	4	4	4	4	4	2	4	1	1	4	1	1	4	3	4	4	4	4

	4	4	4	4	4	5	4	4	4	4	4	4	2	1	1	2	1	1	2
	2	1	9	2	2	1	1	27											
49	4	4	2	2	4	4	4	3	2	4	2	2	1	5	4	4	4	4	4
	4	4	4	3	4	3	2	2	2	1	3	1	2	2	2	2	1	4	4
	4	4	4	4	3	4	3	4	3	4	4	4	2	2	2	4	1	4	2
	1	2	9	3	2	1	1	27											
50	5	1	1	1	5	2	2	2	2	4	3	1	2	4	3	3	3	4	4
	4	5	4	5	4	4	2	4	1	1	3	1	1	1	2	2	3	4	5
	5	5	5	5	3	4	4	3	2	5	4	5	4	2	4	3	1	3	1
	1	3	1	4	2	1	1	30											
51	5	2	3	2	5	2	2	2	1	4	2	2	2	4	4	4	4	3	4
	4	3	3	4	3	4	3	4	5	2	3	2	2	3	4	3	4	4	4
	4	4	4	3	3	3	4	3	3	4	4	5	4	1	1	3	1	4	2
	1	1	9	4	2	1	1	27											
52	4	2	2	4	2	4	3	3	3	4	3	3	2	4	4	3	4	4	4
	3	3	3	4	3	3	3	3	3	3	4	2	1	2	2	1	1	4	4
	4	4	4	4	4	4	4	3	3	4	4	4	2	2	2	3	3	2	2
	1	1	9	4	1	1	1	27											
53	5	4	3	2	3	1	4	4	2	2	2	2	3	4	2	3	4	3	2
	4	4	3	4	4	2	3	3	2	1	2	1	2	2	3	3	2	4	4
	5	5	4	4	3	2	3	4	3	4	3	3	1	1	1	3	1	3	1
	1	3	1	4	1	1	1	33											
54	4	1	4	2	3	2	1	4	2	5	2	2	2	4	4	4	3	3	3
	4	4	5	4	4	4	4	3	4	2	4	1	2	3	5	5	1	4	4

	5	5	5	5	3	4	2	5	4	4	4	4	1	1	3	3	1	4	1
	1	2	9	4	3	1	1	27											
55	5	2	4	2	4	5	4	5	4	5	1	1	2	4	1	4	5	5	4
	2	4	4	4	5	3	1	2	2	1	2	1	1	1	4	1	1	5	5
	5	5	5	5	5	3	2	1	2	5	5	5	3	5	5	5	2	2	1
	1	1	9	2	2	1	1	26											
56	5	3	3	2	3	2	3	4	2	3	1	2	1	4	5	4	4	5	3
	4	5	3	5	4	1	2	4	2	1	2	1	2	4	2	4	3	4	5
	3	4	5	3	3	2	2	3	2	4	4	5	1	3	1	3	1	4	2
	1	3	2	3	1	1	3	28											
57	5	2	4	2	4	1	1	2	1	5	4	3	3	3	4	4	4	5	5
	4	5	5	5	4	4	3	3	3	3	3	1	2	2	3	3	1	3	4
	4	4	5	3	4	4	5	5	3	4	5	5	2	1	2	3	1	4	2
	1	3	9	3	2	1	1	30											
58	4	2	3	2	4	3	3	3	3	4	2	2	2	5	3	4	4	4	3
	5	5	4	4	3	2	4	2	3	4	4	2	4	3	2	3	1	3	4
	5	4	5	3	4	5	5	5	5	4	5	3	1	2	1	3	1	3	2
	1	2	9	3	2	1	2	29											
59	4	2	4	2	2	3	4	4	3	2	1	2	2	4	2	3	3	3	3
	4	3	4	4	3	2	2	3	2	2	3	1	1	3	4	3	3	3	4
	4	4	4	3	2	3	2	3	1	3	4	3	1	1	1	3	1	2	2
	1	3	9	4	2	1	1	27											
60	4	2	4	2	3	3	3	3	2	4	1	1	1	4	3	4	4	4	4
	4	4	4	4	3	3	3	3	3	2	3	2	2	1	1	2	1	3	3

	4	4	5	5	4	4	4	5	4	4	4	3	2	2	2	3	1	4	2
	1	3	9	3	2	1	1	30											
61	5	1	5	1	5	2	2	3	3	2	4	4	3	5	4	5	5	5	4
	5	5	5	5	5	1	1	4	3	1	2	1	1	1	4	2	3	3	5
	5	5	4	5	3	3	4	2	1	5	4	5	3	1	2	4	1	4	1
	1	1	9	5	2	1	2	27											
62	4	2	3	3	3	3	4	4	2	3	4	2	4	5	3	5	4	4	3
	4	4	5	4	3	2	2	3	2	1	3	1	1	4	4	4	3	4	5
	5	5	5	3	4	3	1	4	2	4	4	4	5	3	1	3	1	2	2
	1	3	1	2	2	1	3	-99											
63	4	2	4	3	3	2	3	3	3	4	2	2	3	3	4	4	4	4	5
	4	3	3	5	4	2	3	2	2	3	3	1	3	2	2	2	2	3	3
	4	3	2	2	3	4	2	4	2	3	3	3	2	1	2	4	2	3	1
	1	2	0	3	2	1	1	-99											
64	4	2	4	2	3	2	4	5	3	2	1	1	1	5	3	4	5	4	2
	4	5	3	5	5	2	1	1	2	2	2	1	1	3	5	3	2	3	3
	4	5	5	4	1	2	4	3	2	3	5	3	3	1	1	3	1	4	2
	1	1	0	3	3	1	1	-99											
65	2	5	2	5	2	5	4	3	3	4	2	2	2	4	4	3	4	4	2
	4	4	2	4	2	2	2	4	2	2	2	1	2	4	3	2	2	4	4
	4	4	4	4	3	2	2	1	2	3	3	4	1	3	1	2	1	2	2
	1	1	0	3	1	3	2	-99											
66	4	3	2	3	2	4	2	2	2	4	2	2	4	4	3	3	4	2	3
	3	3	3	4	4	3	4	2	3	2	3	1	2	3	3	3	3	3	4

	3	4	4	3	3	4	4	3	3	3	3	4	1	1	1	4	2	3	1
	1	1	0	5	2	1	1	-99											
67	3	3	3	5	3	4	4	3	3	3	1	1	1	5	3	4	4	4	4
	5	5	4	5	4	3	2	3	4	3	3	2	2	3	4	4	4	4	4
	3	3	5	4	4	4	4	4	4	4	4	4	1	4	1	4	5	4	2
	1	2	0	4	1	1	1	-99											
68	3	2	3	2	3	3	2	2	2	3	1	1	1	4	4	4	4	4	4
	4	4	4	4	4	3	3	3	3	2	2	2	2	3	4	3	3	4	5
	5	5	4	4	4	4	4	3	3	4	4	5	3	4	2	4	2	3	2
		1	0	4	2	3	3	-99											
69	3	2	2	2	3	3	1	1	1	3	1	2	1	3	2	4	4	4	4
	4	4	4	2	3	4	4	3	3	4	4	3	4	3	3	3	2	4	4
	4	4	4	3	4	4	4	1	3	4	5	5	4	4	1	3	1	4	2
	1	1	0	3	1	3	2	-99											
70	4	4	2	4	2	4	2	2	2	4	1	1	1	5	3	4	4	4	5
	5	5	4	4	4	2	2	1	1	3	3	2	2	3	3	3	2	3	2
	3	5	4	3	3	5	5	4	3	2	5	4	3	2	4	4	1	1	2
	1	1	0	4	2	5	1	-99											
71	5	1	4	2	5	2	1	3	2	3	2	1	2	2	2	3	4	5	5
	4	5	4	5	5	3	4	5	1	3	3	2	3	1	2	2	2	3	3
	4	4	5	2	4	5	3	5	4	4	4	3	5	4	2	2	1	4	1
	1	2	0	5	2	1	1	-99											
72	5	4	5	1	5	1	1	3	1	4	4	5	1	5	5	4	5	5	5
	4	5	3	4	5	4	5	5	5	2	4	3	2	2	2	2	3	5	5

	5	5	5	4	5	4	5	4	4	5	5	5	3	4	1	3	1	2	1
	1	1	0	3	2	3	2	-99											
73	3	3	3	3	3	3	3	4	3	2	3	3	3	3	2	4	5	4	4
	5	5	4	4	4	2	1	2	2	1	2	1	2	2	3	3	3	4	5
	5	5	5	4	3	3	2	2	2	3	3	4	2	4	1	3	1	3	1
	1	2	0	4	2	3	2	-99											
74	1	4	2	4	2	4	4	4		2	4	4	3	3	2	3	4	2	2
	4	3	2	3	3	5	1	3	2	1	2	1	1	4	4	4	4	2	2
	2	1	3	2	2	2	1	3	2	1	3	2	1	3	1	3	1	3	2
	1	2	0	3	1	3	3	-99											
75	2	4	2	2	2	2	3	3	2	3	4	2	2	5	4	4	5	4	3
	5	5	4	5	3	2	2	3	2	2	2	2	1	3	5	4	3	3	4
	5	4	5	4	3	3	4	4	2	3	5	3	3	3	4	5	2	4	2
	1	2	0	5	2	1	1	-99											
76	4	2	4	2	3	2	4	4	4	2	4	4	3	2	2	4	5	3	3
	3	4	4	4	4	4	2	3	2	2	2	1	1	1	4	3	3	3	5
	5	5	5	3	2	2	2	2	2	4	4	3	3	3	1	4	1	2	2
	1	2	0	5	2	1	1	-99											
77	2	3	3	4	4	4	2	3	2	1	4	3	3	4	2	4	3	4	4
	4	4	4	3	3	2	4	3	3	2	4	1	3	2	1	2	1	3	4
	2	3	4	4	4	3	1	4	4	4	4	4	2	1	1	3	3	2	2
	1	2	0	4	2		1	-99											
78	2	4	2	4	4	3	4	5	4	3	1		2	3	3	3	4	4	2
	3	4	3	5	3	4	3	3	2	2	3	1	1	1	3	3	2	3	4

	4	4	4	3	2	2	3	4	3	4	4	4	3	2	2	3	1	3	1
	1	2	1	2	2	1	1	-99											
79	4	2	3	2	3	3	3	3	3	3	2	3	2	4	4	4	4	4	4
	4	4	3	4	4	3	2	3	3	3	4	1	2	3	3	4	3	4	4
	4	4	4	3	3	4	4	3	2	4	4	4	2	3	3	3	1	3	1
	1	1	0	4	2	2	2	-99											
80	2	5	2	5	2	5	5	5	5	3	4	5	3	4	2	5	5	5	5
	5	5	4	4	4	2	2	3	3	3	2	1	2	2	2	2	2	2	5
	4	5	4	2	2	2	4	3	2	4	5	4	1	1	1	4	1	3	2
	1	2	0	2	2	1	1	-99											
81	5	2	5	2	5	2	3	4	3	3	2	2	2	3	3	4	4	5	4
	5	5	4	5	4	4	4	4	3	3	3	1	3	3	2	2	2	3	4
	3	4	4	4	4	4	4	4	3	4	5	3	4	3	1	3	1	5	2
	1	1	0	4	1	1	1	-99											
82	4	1	4	1	3	2	4	4	3	4	4	3	3	2	3	4	4	4	4
	3	4	4	4	4	3	3	4	3	2	3	1	2	3	2	4	4	4	1
	4	2	3	4	3	4	4	3	3	3	4	3	4	1	1	4	1	3	2
		1		3	2	5	1	-99											
83	4	2	4	2	4	2	2	2	2	3	4	3	3	3	3	4	3	4	5
	3	4	4	5	4	3	3	3	3	3	3	1	3	2	2	2	2	4	4
	4	4	4	3	3	4	5	4	3	4	4	4	4	2	3	3	1	4	1
	1	1	0	5	2	1	1	-99											
84	3	4	4	3	4	3	2	3	2	3	2	2	1	3	2	3	3	4	5
	4	5		5	5	4	4	3	1	1	3	1	1	1	5	4	4	3	3

	4	5	4	4	1	5	4	5	5	5	5	4	1	1	1	1	1	4	1
	1	1	0	3	2	1	1	-99											
85	4	3	4	2	2	2	2	3	2	4	2	2	2	4	3	4	4	5	4
	5	5	4	4	4	3	1	3	3	1	3	1	1	3	3	3	3	3	4
	4	4	4	4	3	3	2	4	3	4	4	4	2	1	2	3	1	4	2
	1	2	0	3	1	1	1	-99											
86	4	2	4	2	3	2	2	3	2	3	3	3	3	3	3	3	4	3	3
	3	3	4	3	3	4	2	2	2	1	3	1	2	2	2	2	2	3	3
	3	3	3	3	3	3	3	4	3	3	3	3	2	2	2	3	1	2	1
	1	2	0	5	2	1	1	-99											
87	3	3	3	4	3	3	3	3	3	4	2	2	1	4	3	4	4	4	3
	4		4	3	3	4	2	3	2	2	3	1	2	2	4	4	3	3	3
	4	3	3	3	3	3	4	4	3	3	3	3	2	1	1	2	1	3	2
	1	3		3	1	1	1	-99											
88	3	2	3	2	3	3	3	3	2	3	2	3	2	4	3	5	4	4	3
	4	4	4	5	4	4	4	3	3	3	3	4	2	2	2	2	2	4	3
	4	3	4	3	3	4	3	4	2	4	5	3	4	4	1	4	4	3	2
	1	1	0	3	2	1	2	-99											
89	3	4	4	3	2	4	4	3	4	3	2	3	2	4	4	5	5	3	4
	5	5	2	4	3	2	1	2	2	2	2	1	2	2	3	3	4	4	4
	3	3	4	3	2	3	2	4	3	4	4	4	2	2	1	4	1	3	1
	1	1	0	5	2	2	2	-99											
90	4	3	4	3	4	3	2	3	2	4	2	2	2	4	3	5	5	5	3
	5	5	5	5	5	3	2	3	3	1	4	1	2	4	5	5	5	4	4

	4	4	4	4	3	3	1	4	3	4	5	4	2	2	5	3	1	3	2
	1	3		5	1	1	1	-99											
91	3	2	4	3	3	3	2	4	2	3	2	3	2	3	3	4	4	4	4
	4	4	4	4	4	3	3	3	3	2	3	1	3	3	3	4	3	3	3
	3	4	4	3	3	3	4	4	2	3	3	3	2	2	3	3	1	3	2
	1	2	0	4	2	1	1	-99											
92	3	2	3	2	3	4	3	4	2	4	2	2	1	3	3	4	5	4	4
	5	5	5	4	4	2	2	3	2	1	3	1	1	2	3	3	2	3	4
	5	4	4	4	3	3	1	3	3	4	3	4	4	1	1	3	1	1	2
	1	2	0	4	1	1	1	-99											
93	4	3	4	3	3	3	3	4	4	4	3	3	2	4	3	3	5	4	2
	5	4	2	3	4	2	1	2	1	1	1	1	1	1	2	1	1	4	5
	4	3	4	2	1	1	1	1	1	3	3	4	2	1	1	3	3	1	2
	1	2	0	3	1	1	1	-99											
94	3	2	4	2	4	4	2	2	2	3	2	3	2	3	3	4	4	4	3
	3	4	5	4	4	3	3	3	3	3	3	2	3	2	4	3	3	3	4
	4	4	4	3	3	3	3	4	3	4	4	4	3	2	3	4	1	3	1
	2	2	0	4	2	1	1	-99											
95	5	1	5	1	5	1	3	2	2	5	2	2	1	4	4	4	4	4	5
	5	5	4	5	4	3	5	4	5	3	4	4	5	4	5	4	3	5	5
	5	5	5	5	5	5	5	5	5	5	5	5	5	2	4	4	1	5	1
	1	1	0		3	1	1	-99											
