# Supplementary material for: The associations between resilience, self-care, and burnout among medical students
Source: PLoS One. 2024 Sep 19;19(9):e0309994. doi: 10.1371/journal.pone.0309994 (PMC11412677; doi:10.1371/journal.pone.0309994)
Supplement: Attachment — Submitted filename: PONE-Response to Reviewers.docx [file pone.0309994.s003.docx]

22.07.24

Dear Professor Douglas Aninng Opoku, MPH
Academic Editor
PLOS ONE

Thank you for the opportunity to improve our manuscript (PONE-D-24-16703) titled: "The Associations Between Resilience, Self-Care, and Burnout among Medical Students" and resubmit it to PLOS ONE journal.

We addressed the editor and reviewers' important and helpful comments.

This letter includes the comments followed by a reply to each and––when relevant––the text from the revised version in *Italics.*

We attached two new versions of the manuscript: one with Track for Changes and a clean one. Additionally, we added a statement regarding the financial disclosure within the cover letter, and re-uploaded a supplementary file.

We appreciate this review process and hope that you will find our revised version suitable for publication in the distinguished PLOS ONE journal.

**Journal requirements:**

Comment 1

When submitting your revision, we need you to address these additional requirements.

Please ensure that your manuscript meets PLOS ONE's style requirements, including those for file naming. The PLOS ONE style templates can be found at 
<https://journals.plos.org/plosone/s/file?id=wjVg/PLOSOne_formatting_sample_main_body.pdf> and 
<https://journals.plos.org/plosone/s/file?id=ba62/PLOSOne_formatting_sample_title_authors_affiliations.pdf>.

Reply 1

As requested, we ensured that the style requirements of PLOS ONE are used. We verified this throughout the entire manuscript (i.e., headings, tables, figures, references etc.).

Comment 2

Please ensure that you include a title page within your main document. We do appreciate that you have a title page document uploaded as a separate file, however, as per our author guidelines (<http://journals.plos.org/plosone/s/submission-guidelines#loc-title-page>) we do require this to be part of the manuscript file itself and not uploaded separately.
Could you therefore please include the title page into the beginning of your manuscript file itself, listing all authors and affiliations.

Reply 2

As suggested, we included the title page into the beginning of our manuscript file, listing all authors and affiliations carefully following the guidelines.

Comment 3

Thank you for stating the following financial disclosure: 
 [THE ISRAEL NATIONAL INSTITUTE FOR HEALTH POLICY RESEARCH partially supported the study.].  
Please state what role the funders took in the study. If the funders had no role, please state: "The funders had no role in study design, data collection and analysis, decision to publish, or preparation of the manuscript." 
If this statement is not correct you must amend it as needed. 
Please include this amended Role of Funder statement in your cover letter; we will change the online submission form on your behalf.

Reply 3

We added the role of the funders to the cover letter file, using the suggested statement.

Comment 4

Please include your full ethics statement in the ‘Methods’ section of your manuscript file. In your statement, please include the full name of the IRB or ethics committee who approved or waived your study, as well as whether or not you obtained informed written or verbal consent. If consent was waived for your study, please include this information in your statement as well.

Reply 4

We added the university name (i.e., the full name of the ethics committee), as well as the approval number (#6116). The use of written informed consent is mentioned:

*"The Tel Aviv University Ethics Committee approved the study (approval #6116), and all participants signed a written informed consent form before completing the self-reported questionnaires."*

Comment 5

Please include your tables as part of your main manuscript and remove the individual files. Please note that supplementary tables (should remain/ be uploaded) as separate ""supporting information"" files.

Reply 5

We now included all the tables in the main file.

Comment 6

We are unable to open your Supporting Information file [self care-PO.sav]. Please kindly revise as necessary and re-upload.

Reply 6

We apologize. We now have copied the data, saved it as a PDF file, and re-uploaded it.

Comment 7

Please include captions for your Supporting Information files at the end of your manuscript, and update any in-text citations to match accordingly. Please see our Supporting Information guidelines for more information: <http://journals.plos.org/plosone/s/supporting-information>.

Reply 7

Thank you for this guidance. We have done this now, page 8:

*"(See Table 1 for participants' demographic characteristics and S1File for the study fundamental data)."*

Comment 8

Please review your reference list to ensure that it is complete and correct. If you have cited papers that have been retracted, please include the rationale for doing so in the manuscript text, or remove these references and replace them with relevant current references. Any changes to the reference list should be mentioned in the rebuttal letter that accompanies your revised manuscript. If you need to cite a retracted article, indicate the article’s retracted status in the References list and also include a citation and full reference for the retraction notice.
Reply 8

We reviewed the reference list and changed it to Vancouver Style. Moreover, in the original version we forgot to put reference 31 in the list. We added it in the revised version.

Comments from PLOS Editorial Office:

We note that one or more reviewers has recommended that you cite specific previously published works. As always, we recommend that you please review and evaluate the requested works to determine whether they are relevant and should be cited. It is not a requirement to cite these works. We appreciate your attention to this request.

Reply

The specific reference was relevant to our manuscript. Hence, we added it (Page 2 and Page 7).

**Reviewer #1**
Comment 1

I read your work with great interest, and I am pleased to congratulate you on your contribution to scientific research.
I believe that the article is novel and interesting, that it has a sufficient impact, and that it adds to the knowledge base. Plagiarism was not detected. The study appears to follow relevant guidelines and provides an original contribution to the existing scientific literature. There are no flaws in the data presented, and there are no misleading or false conclusions.

Reply 1

Thank you very much for the supportive feedback and finding the manuscript a valuable contribution to the field.

Comment 2
The current study is scientifically valid. The reasons for performing the study are clear. However, before proceeding with its publication, it is necessary to make some minor revisions. I strongly suggest that the authors highlight that resilience has been proven to be key to the mental health and well-being of healthcare workers (please cite: Safiye, T., Vukčević, B., Čabarkapa, M. (2021). Resilience as a moderator in the relationship between burnout and subjective well-being among medical workers in Serbia during the COVID-19 pandemic. Vojnosanitetski Pregled, 78 (11), 1207–1213.).
Reply 2

Thank you for the relevant reference. As suggested, we added it, in two places: Page 2: *"One personal ability that may protect individuals from burnout is resilience [13]."*

And Page 7: *"Studies have addressed the resilience–burnout relationship (e.g., [13])…"*

Comment 3
I recommend the article for publication in PLOS ONE after minor revisions.
Reply 3

Thank you very much for the recommendation and guidance!

**Reviewer #2**

Comment 1

Summary: A well written manuscript impressive flow of thought and written in intelligible fashion.

Reply 1

Thank you very much for the supportive feedback.

Comment 2

Results: Table 1: the columns do not correspond correctly with the other categories in the rows. Please see highlighted portions of the table
Reply 2

To enhance clarity, we added borders to the table to clarify the fit between categories and rows. Moreover, in one variable (religion), there was one missing data. Hence, the "Frequency" sums in 94 (rather than N=95). The valid percentages are calculated accordingly. We added this information to the column title and noted it under Table 1.

Comment 3

Introduction and methods: some highlighted portions require rephrasing
Reply 3

We rephrased the highlighted sentences:

- Page 2: "*Burnout is common in members of the healthcare professions, including nurses, physicians, and medical students, who are involved in caregiving and intensive interaction with people, often with a tendency to put others in the center, sometimes comprising their own well-being (Nkabinde-Thamae et al., 2022)*."
- Page 8: *"A quantitative cross-sectional study was conducted among a class of 95 fourth-year medical students from Tel Aviv University in Israel (M_age_=27.26, SD=2.81), representing a response rate of 76% (95 of 125 students completed the questionnaires). Data from these students were collected in May 2016, immediately after a 14-week internal medicine clerkship. The clerkship is a critical and very intense stage in students' training, requiring them to adjust to the medical system. During this period, medical students may experience increased stress and distress (Dyrbye et al., 2006)."*

Comment 4
Data which was used for the regression analysis was not accessed by me.

Reply 4

We apologize that the SPSS "Sav" file was not accessible. We copied all the numbers to a PDF file and uploaded it.

Comment 5

Conclusion: 'The present study reveals that strengthening medical students' resilience levels is related to encouraging self-care actions (stress management, spiritual growth, and interpersonal relations) and is, in turn, related to a lower level of burnout (emotional exhaustion and personal accomplishment).'
comment: Given the study design it is difficult to agree with this comment.
Reply 5

As suggested, we rephrased this sentence, Page 19:

*"The present study suggests that medical students' high levels of resilience are associated with taking more self-care actions (stress management, spiritual growth, and interpersonal relations). Additionally, taking more self-care actions is associated with lower levels of burnout (emotional exhaustion and personal accomplishment)*.*"*

Comment 6

References 1-4 should be written in full in consistency with all other references.

Reply 6

We used the short version to keep our anonymity. We added all necessary information. Now these are references 49, 62, 69, and 70.

**We greatly appreciate the editor and reviewers' comments and guidance.**
