# Supplementary material for: The associations between resilience, self-care, and burnout among medical students
Source: PLoS One. 2024 Sep 19;19(9):e0309994. doi: 10.1371/journal.pone.0309994 (PMC11412677; doi:10.1371/journal.pone.0309994)
Supplement: Attachment — Submitted filename: Response to Reviewers.docx [file pone.0309994.s005.docx]

21.08.2024

Dear Professor Douglas Aninng Opoku, MPH
Academic Editor
PLOS ONE

Thank you for the opportunity to revise and resubmit our manuscript: “The Associations Between Resilience, Self-Care, and Burnout among Medical Students” (PONE-D-24-16703R1) to PLOS ONE journal. We greatly appreciate the time and effort you and the reviewers invested in evaluating our manuscript and providing constructive feedback.

We are pleased that the reviewers responded positively to our revisions in response to their previous comments. We followed Reviewer 2 grammatical concerns and corrected our manuscript accordingly.

We appreciate the guidance and hope the revised version meets the standards required for publication in PLOS ONE journal.

Sincerely,
Keren Michael, Dana Schujovitzky, and Orit Karnieli-Miller

**Reviewer #1:**

Dear Authors,
The manuscript has been improved, and I recommend that the paper be accepted without any changes and published in the journal PLOS ONE.
Best regards

**Reply:**

Thank you very much for your recommendation to accept the manuscript.

**Reviewer #2:**

The statistical analysis seems to have been performed appropriately however I did not replicate with the data to determine the accuracy.
Suggested Corrections:
Introduction: Page 2, line 8 – “comprising their own well-being” should read “compromising their own well- being”
Page 3 line 11 - “underlying” can be better put: suggestion: “underpinning”
Discussion: Page 15, lines 12 and 13: “with whom the present study participants had interpersonal relationships” should be rephrased: suggestion: “whom the study participants had interpersonal relationships with”
Page 18, line 10 “in their stressful lives, will include”, suggestion: “during stressful situations will adopt”

**Reply:**

Thank you for highlighting these corrections! Based on your recommendation we corrected all four sentences as suggested (Page 2, Page 3, Page 15, and Page 18).
